# Biomechanics of knee osteoarthritis from local knee loading to whole kinetic chain imbalance: a narrative review

**DOI:** 10.3389/fbioe.2026.1932171

**Published:** 2026-10-01

**Authors:** Junyang Tang, Wenhai Zhao, Binghua Zhang

**Affiliations:** 1 Affiliated Hospital of Changchun University of Chinese Medicine, Changchun University of Chinese Medicine, Changchun, Jilin, China; 2 Hebei Provincial Hospital of Chinese Medicine, Shijiazhuang, China; 3 Hebei Key Laboratory of Turbidity Toxin Syndrome, Shijiazhuang, China

**Keywords:** biomechanics, global alignment, knee osteoarthritis, local loading, mechanical imbalance

## Abstract

The onset and progression of knee osteoarthritis (KOA) are closely associated with abnormal mechanical loading. Previous research has focused largely on local biomechanical factors, such as lower-limb alignment and the knee adduction moment; however, these factors alone may not fully explain the discordance between radiographic degeneration and clinical manifestations such as pain and functional impairment. From a whole-kinetic-chain perspective, the knee functions as an intermediate link within the continuous spine-to-foot chain. Multisegmental malalignment involving the lumbar spine, pelvis, hip, and ankle-foot complex may shift the center of mass and alter the transmission of ground reaction forces, potentially interacting bidirectionally with local knee malalignment to redistribute joint loads and contribute to cartilage damage and pain. By examining the limitations of knee-centered biomechanical studies, we aim to synthesize evidence on the relationship between global alignment and KOA, explores how global-local load interactions may contribute to KOA progression, and proposes a conceptual framework in which the interplay between global and local malalignment may amplify mechano-inflammation and thereby promote disease progression. This review aims to provide new insights into biomechanical phenotyping, progression-risk assessment, and individualized mechanical interventions for KOA.

## 1 Introduction

KOA is one of the most common chronic degenerative joint diseases among middle-aged and older adults and is a major cause of knee pain, stiffness, mobility limitations, and reduced quality of life (Sharma, 2021). With population aging, the increasing prevalence of obesity, and the cumulative burden of sports-related injuries, the disease burden of KOA continues to rise, making it an important public health concern associated with substantial functional impairment and healthcare utilization (GBD 2021 Osteoarthritis Collaborators, 2023). The pathological changes in KOA are not confined to articular cartilage degeneration but involve multiple joint tissues and processes, including subchondral bone remodeling, synovial inflammation, meniscal degeneration, ligamentous laxity, and periarticular muscle dysfunction (Poole, 2012). Accordingly, KOA is no longer regarded simply as a disease of “cartilage wear” but is increasingly recognized as a whole-joint disorder arising from interactions among mechanical, inflammatory, metabolic, and age-related factors (Bennell et al., 2011; Felson et al., 2016; Kanthawang et al., 2021).

Biomechanical abnormalities have long been recognized as important contributors to the onset and progression of KOA (Mündermann et al., 2024). Previous research has focused largely on local biomechanical alterations within the knee, such as lower-limb malalignment, varus or valgus deformity, increased knee adduction moment (KAM), varus thrust (VT), and uneven load distribution between the medial and lateral compartments (Sharma et al., 2010; Kutzner et al., 2013). This body of work has substantially advanced our understanding of the relationships among abnormal joint loading, cartilage degeneration, joint-space narrowing, and disease progression. However, biomechanical explanations confined to the knee alone may not fully account for the clinical heterogeneity of KOA (Miyazaki et al., 2002; Bennell et al., 2011; Bg et al., 2025). Patients with similar structural changes may exhibit markedly different symptom severity, functional limitations, and rates of disease progression, suggesting that knee-centered biomechanical measures may be insufficient and that broader mechanical influences could also be relevant (Bedson and Croft, 2008; Roemer et al., 2023).

From a musculoskeletal biomechanics perspective, the knee should be viewed not as an isolated load-bearing structure but as an integral component of a continuous kinetic chain involving the spine, pelvis, hip, knee, ankle, and foot (Katsumi et al., 2023; Singh et al., 2025). Evidence suggests that lumbar lordosis, pelvic tilt, and femorotibial torsional alignment are associated with lower-limb mechanical alignment and knee load distribution (Fu et al., 2023; Yoon and Moon, 2024; Tomay Aksoy and Bütün, 2025). During standing and walking, complex compensatory relationships exist within the spine-pelvis-lower-extremity kinetic chain; malalignment or functional impairment at any segment may shift the body’s center of mass, alter ground reaction force trajectories, and redistribute joint moments, potentially reshaping the mechanical microenvironment of intra-articular tissues (Wang et al., 2016; Katsumi et al., 2023; Du et al., 2024; Zhang et al., 2026). Therefore, KOA may reflect not only local knee malalignment but also the cumulative effects of biomechanical imbalance across the whole kinetic chain.

Altered knee loading resulting from local changes or global malalignment may not only contribute to tissue damage but also induce intra-articular inflammatory responses (Sanchez-Adams et al., 2014; Hodgkinson et al., 2022). Emerging evidence from mechanobiology indicates that chondrocytes and other joint-resident cells possess sophisticated mechanosensory systems, including Piezo ion channels, transient receptor potential vanilloid 4 (TRPV4), integrin/focal adhesion kinase complexes, primary cilia, cytoskeletal networks, and YAP/TAZ signaling pathways (Wang N. et al., 2023; Zhang et al., 2025b; Brylka et al., 2024; Nims et al., 2024; Wang et al., 2022). These systems enable cells to sense external mechanical stimuli and translate them into intracellular biochemical signals (Jia et al., 2023). Physiological mechanical stimulation contributes to the maintenance of cartilage homeostasis, whereas excessive, abnormal, or chronic mechanical stress may trigger Ca^2+^ signaling, activate nuclear factor kappa-B (NF-κB) and Mitogen Activated Protein Kinases (MAPK) pathways, activate NOD-like receptor family pyrin domain containing 3 (NLRP3) inflammasome, upregulate the COX-2/PGE_2_ axis, and induce mitochondrial dysfunction, oxidative stress, and extracellular matrix degradation (Batarfi et al., 2025; Liu et al., 2025). Thus, abnormal mechanical stimuli may promote catabolic activation and inflammatory responses, providing a potential mechanistic bridge between biomechanical abnormalities and KOA progression (Jahn et al., 2024).

Building on recent advances in local knee biomechanics, we examine evidence linking global misalignment to KOA and explore potential pathways through which global and local malalignment may alter regional knee loading and contribute to disease progression. We propose a conceptual framework in which global and local malalignment may induce abnormal knee loading, thereby amplifying mechano-inflammation and potentially promoting KOA progression. This framework aims to offer new insights into the biomechanical phenotyping of KOA, progression-risk assessment, and the optimization of individualized mechanical intervention strategies.

## 2 Methods

This study was conducted as a narrative review. A literature search was performed in PubMed from database inception to July, 2026. The search strategy used combinations of keywords and subject terms related to knee osteoarthritis, biomechanics, knee joint loading, malalignment, knee adduction moment, VT, spine, pelvis, hip, ankle, foot, kinetic chain, mechanotransduction, and mechanoinflammation. The reference lists of relevant reviews and key original studies were also screened to identify additional eligible publications.

Studies were considered relevant if they addressed at least one of the following domains: local knee alignment and loading, gait biomechanics, alignment of the spine, pelvis, and lower extremities, the influence of the hip or ankle and foot on knee joint loading, biomechanical compensation across the kinetic chain, mechanotransduction, mechanoinflammation, cartilage or subchondral bone responses to abnormal loading, or the clinical implications of biomechanical assessment in knee osteoarthritis.

The literature search and screening were conducted by two investigators. One investigator performed the initial search and screening, and the second investigator reviewed the selected studies for relevance and completeness. Both human and experimental studies were considered. Human studies were prioritized for evaluating relationships among clinical characteristics, imaging findings, alignment, gait, and joint loading. Animal and *in vitro* studies were included primarily to support mechanistic interpretations related to mechanosensing, intracellular signaling, inflammation, cartilage degradation, and subchondral bone remodeling. Because these study types represent different levels of biological organization and differ in their clinical generalizability, their findings were interpreted separately and were not pooled.


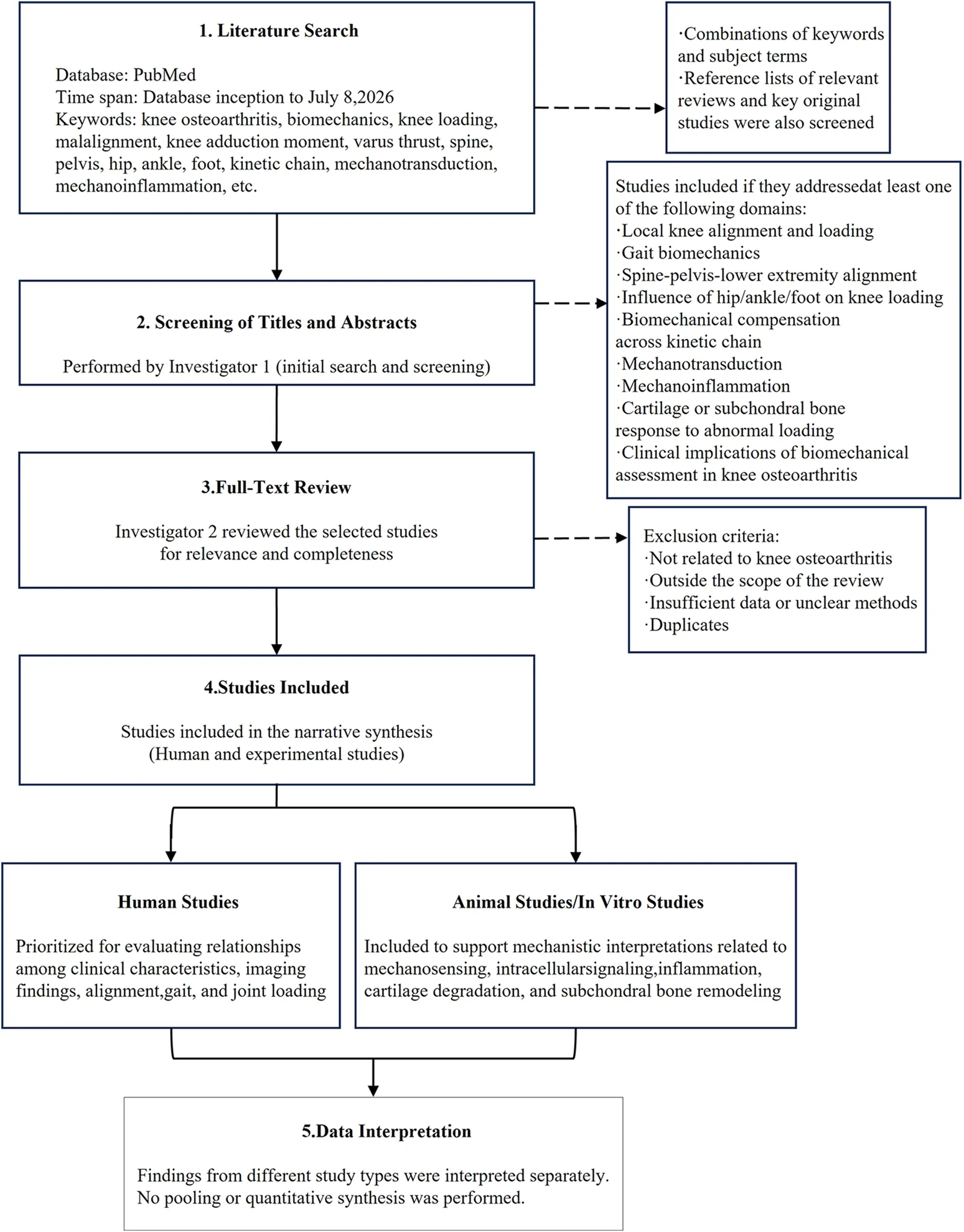


The methodology flow chart was manually drawn by the authors using Microsoft PowerPoint for Microsoft 365, Version 9, on Windows 11.

## 3 Knee osteoarthritis from the perspective of local mechanics

Local biomechanical assessment of KOA encompasses both static alignment and dynamic loading. For static assessment, weight-bearing radiographs are commonly used to quantify parameters such as the hip-knee-ankle angle (HKA) and mechanical axis deviation, thereby characterizing the overall mechanical axis and coronal alignment of the lower limb (Sharma et al., 2001; Marques Luís and Varatojo, 2021; Michalska-Foryszewska et al., 2024). In addition, the distal femoral valgus angle, proximal tibial varus angle, and joint-line obliquity are commonly used to further localize deformity to the femoral, tibial, or joint-line level (Zhang H. et al., 2023; Pratobevera et al., 2024). These radiographic indices provide important information for examining the relationship between coronal-plane malalignment and load distribution across the medial and lateral compartments of the knee.

Dynamic assessment encompasses both external surrogate measures and model-based estimates of internal joint loading. Among external approaches, three-dimensional gait analysis is widely used (Nishino et al., 2015). Because varus deformity is common in KOA, the KAM, which is widely used as an external surrogate of medial compartment loading during gait, and VT have been extensively studied (Chang et al., 2010; Kutzner et al., 2013) (Figure 1). The first and second KAM peaks and the KAM impulse characterize medial loading during early stance, late stance, and the entire stance phase, respectively (Kutzner et al., 2013; Paterson et al., 2017; Asay et al., 2018). Recent studies further suggest that dynamic biomechanical alterations in patients with KOA are not confined to the coronal plane; the knee flexion moment, rotational moment, total knee moment, and relative contributions of different moment components during the gait cycle may vary with disease severity (Asay et al., 2018; Ulrich et al., 2023). Notably, in early KOA, an increased relative contribution of KAM may precede obvious static varus deformity, suggesting that dynamic loading abnormalities might reflect local mechanical imbalance earlier than conventional radiographic alignment changes (Suzuki et al., 2023). Although the knee adduction moment, or KAM, is commonly used as a surrogate measure of medial tibiofemoral loading, it is an external frontal plane moment and does not directly represent joint contact force (Walter et al., 2010). Actual joint loading is also influenced by factors such as muscle co contraction, sagittal plane moments, joint geometry, and individual gait strategies (Manal et al., 2015). Therefore, a reduction in KAM does not necessarily indicate a reduction in medial compartment contact force. KAM is better regarded as an indirect indicator of medial knee loading and should be interpreted in combination with other kinetic measures and musculoskeletal models for a more comprehensive assessment. In addition, bilateral asymmetry should be considered when interpreting dynamic knee loading in KOA. Patients with predominantly unilateral disease may reduce loading of the affected limb and redistribute mechanical demands toward the contralateral limb, resulting in asymmetries in joint loading, muscle forces, and temporal gait characteristics (Gustafson et al., 2019; Liu et al., 2023). Therefore, the contralateral limb should not necessarily be regarded as a normal internal control. Persistent compensatory loading may also contribute to structural or symptomatic changes in the contralateral knee, highlighting the importance of bilateral biomechanical assessment in KOA (Corrigan et al., 2023). VT is another important local biomechanical phenotype of KOA that has received increasing attention (Chang et al., 2013; Sharma et al., 2017). It typically manifests as a sudden increase in varus alignment, or a bowing-out motion of the knee, during the stance phase of gait and may be regarded as a manifestation of dynamic frontal-plane instability arising from the combined effects of static varus alignment, ligamentous laxity, inadequate neuromuscular control, and compensatory foot/ankle mechanics (Misu et al., 2022). VT may more comprehensively capture transient impact loading and abnormal frontal-plane shear conditions in the medial compartment during weight-bearing gait and has been associated with greater medial knee loading, pain, functional limitations, and structural progression in KOA (Wink et al., 2019; Misu et al., 2023). Recent research on VT has gradually shifted from visual gait observation toward more quantitative approaches, including video-based assessment, three-dimensional motion capture, and inertial measurement unit-based analysis (Misu et al., 2023; Di Bacco et al., 2025). However, substantial heterogeneity remains across studies in the definition, measurement methods, and threshold criteria for VT, highlighting the need for a more unified, reproducible, and clinically feasible evaluation system (Di Bacco et al., 2025). In addition to KAM and VT, foot progression angle, step width, walking speed, knee flexion angle, tibial rotation, knee flexion contracture, and quadriceps and hip abductor function have also been incorporated into studies of local knee loading (Baniasad et al., 2022; Thomas et al., 2022; Baghi et al., 2025). Changes in foot progression angle may alter the moment arm of the ground reaction force relative to the knee joint center, thereby affecting peak KAM and load distribution within the medial compartment (Uhlrich et al., 2022). Recent randomized controlled trials suggest that personalized gait-retraining programs targeting foot progression angle may reduce medial knee loading, alleviate pain, and potentially slow structural deterioration (Uhlrich et al., 2025). These findings suggest that local dynamic loading may serve not only as an observational indicator of disease progression but also as a potential target for therapeutic intervention.

**FIGURE 1:**
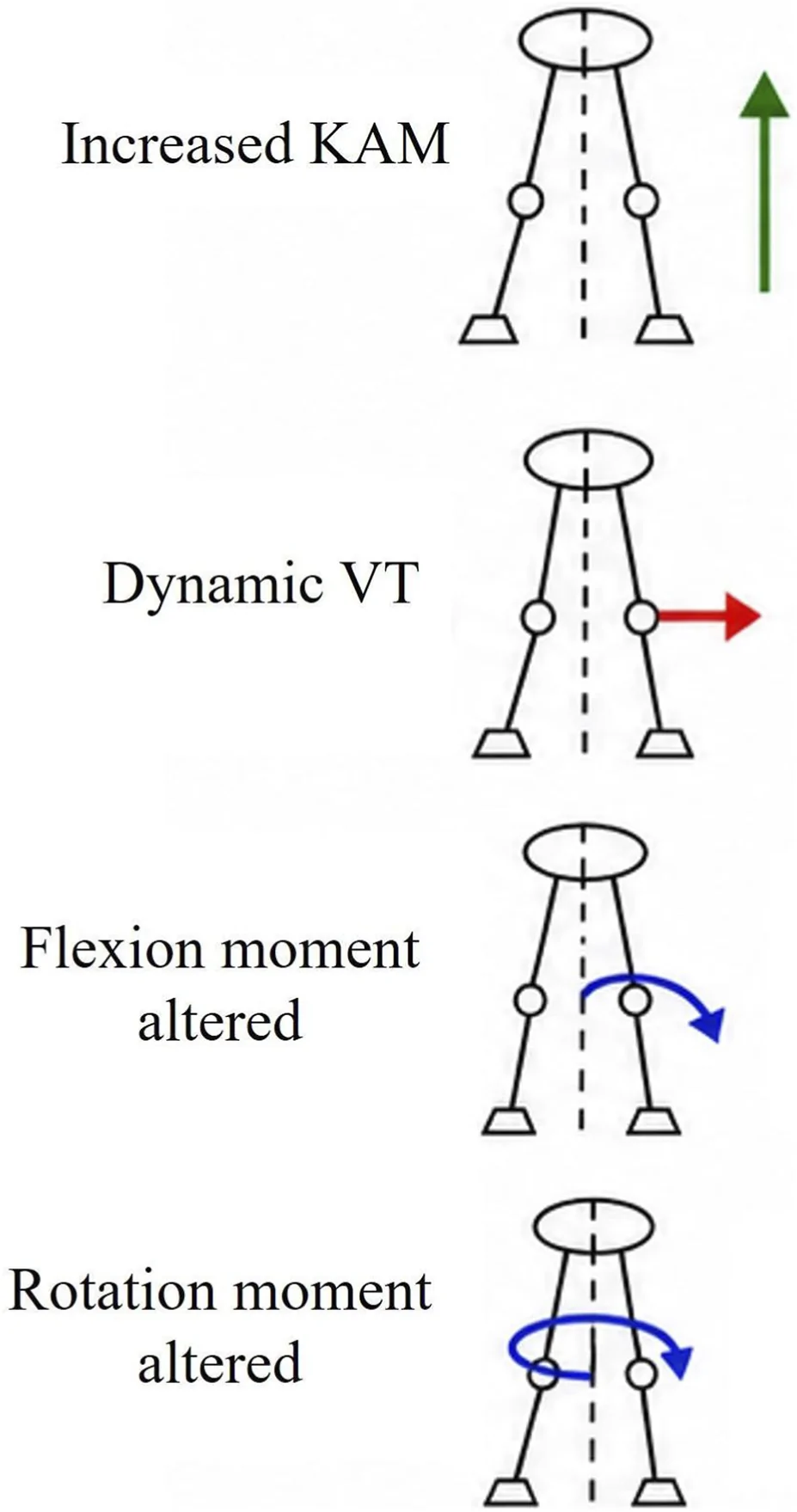
Dynamic loading abnormalities in knee osteoarthritis.

Model-based assessment of internal joint loading integrates gait kinematics, ground reaction forces, and muscle forces using musculoskeletal and finite element models to estimate mechanical variables such as tibiofemoral contact force, contact pressure, cartilage compressive strain, fibril strain, and shear strain (Mononen et al., 2023; Moura et al., 2024; Zhang et al., 2025a). Compared with external surrogate measures such as KAM, these internal mechanical indices may more directly characterize the local mechanical microenvironment of articular cartilage and subchondral bone. Recent studies suggest that patients who develop medial KOA progression may already exhibit higher medial compartment contact pressure and abnormal shifts in contact location at baseline, raising the possibility that internal mechanical indices could serve as sensitive predictors of disease progression (Kutzner et al., 2013).

Moreover, advances in artificial intelligence, wearable sensors, and low-cost motion-analysis technologies are gradually extending local biomechanical assessment of KOA from laboratory settings to clinical practice and daily-life environments (Bacon et al., 2024; Xie et al., 2024). Although traditional three-dimensional gait analysis can accurately capture joint kinematic and kinetic parameters, it typically depends on motion-capture systems, force plates, and specialized inverse-dynamics or musculoskeletal-modeling workflows (Cappozzo et al., 2005). These requirements make such analyses expensive, technically demanding, spatially constrained, and difficult to implement widely in large-scale clinical studies (Snyder et al., 2023; Wipperman et al., 2024). In recent years, studies using inertial measurement units, instrumented insoles, plantar-pressure systems, markerless video-based pose estimation, and machine-learning algorithms have attempted to predict KAM, tibiofemoral contact force, and KOA-related abnormal gait patterns at relatively low cost (Ben Hassine et al., 2024; Li et al., 2024; Matsuda et al., 2025). Biomechanical variables derived from wearable technologies should first be validated against established reference methods, as their accuracy may be influenced by multiple factors, including sensor placement, calibration procedures, soft tissue motion, device algorithms, and individual differences in gait patterns (Kobsar et al., 2020; Kiernan et al., 2024). In addition, real world factors such as walking speed, terrain, footwear, and compensatory movement strategies may substantially affect the measured variables (Micó-Amigo et al., 2023). Therefore, estimates of knee joint loading derived from wearable technologies may not fully or accurately reflect kinetic variables. Before wearable technologies can be routinely implemented for clinical monitoring, standardized validation frameworks, transparent reporting of algorithms, and clinically meaningful thresholds are essential. Although wearable technologies hold considerable promise, their translation from laboratory assessment to routine clinical application requires further investigation.

## 4 Knee osteoarthritis from the perspective of global mechanics

### 4.1 Spinopelvic alignment and KOA

From a whole-body biomechanical perspective, the spinopelvic system plays an upstream role in maintaining sagittal balance and regulating the relationship between the body’s center of mass and the lower extremities (Cho et al., 2023; Knebel et al., 2024). Lumbar lordosis, pelvic tilt, PI-LL mismatch, and anterior or posterior trunk inclination may influence the position of the center of mass and the pathway through which ground reaction forces are transmitted across the lower limbs (Lafage et al., 2023; Balmaceno-Criss et al., 2024). When lumbar lordosis decreases, pelvic retroversion occurs, or PI-LL mismatch develops, the body may recruit compensatory strategies such as hip flexion, knee flexion, and altered ankle posture to maintain upright balance (Fu et al., 2023; Wang J. et al., 2023). Although these compensatory mechanisms may help preserve global postural stability, they may also alter knee flexion posture, tibiofemoral contact location, and compartmental load distribution, potentially exposing the articular cartilage, meniscus, and subchondral bone to abnormal compressive or shear-related mechanical environments over time (Murakami et al., 2025) (Figure 2).

**FIGURE 2:**
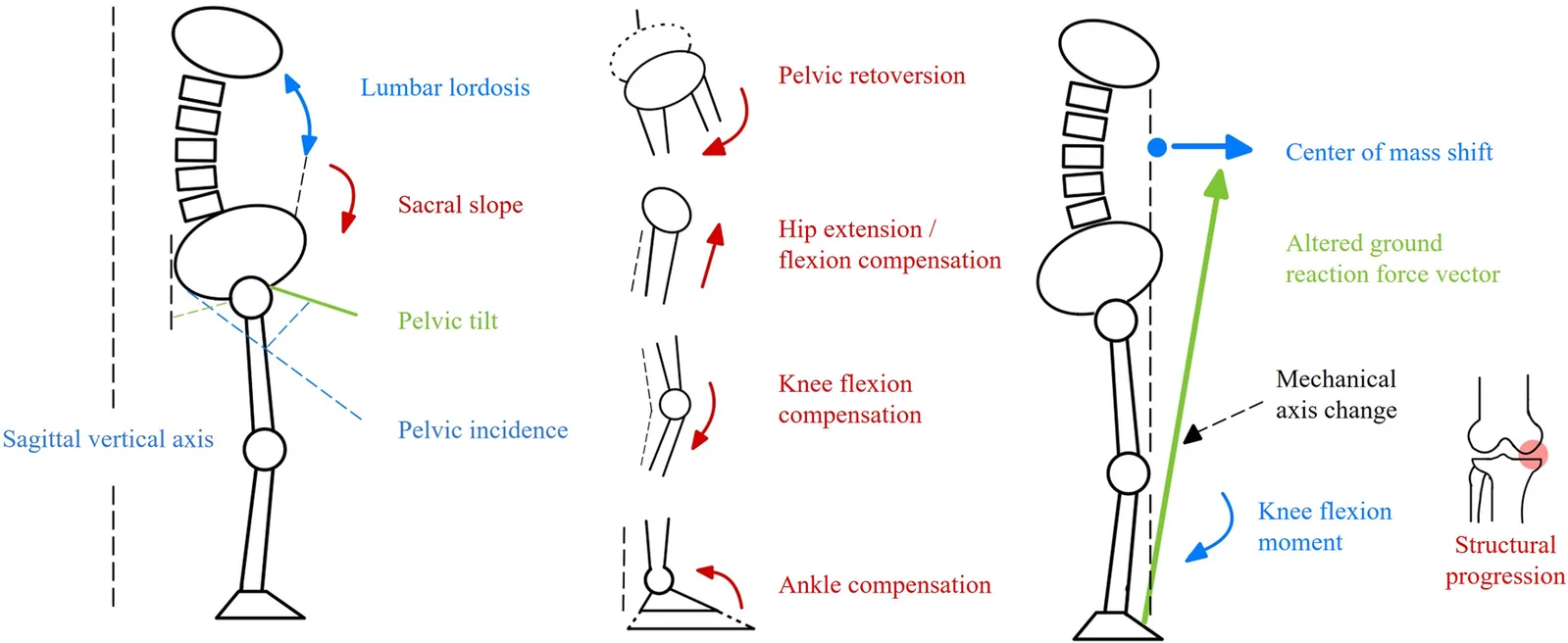
Spinopelvic alignment and knee loading.

### 4.2 Mechanical role of the hip joint and femoral rotation in KOA

Abnormal internal or external hip rotation, hip abductor weakness, and insufficient lateral pelvic stability may increase the tendency toward dynamic knee varus and medial compartment loading (Iijima et al., 2019; Yoon and Moon, 2024; Ambrus et al., 2026). During the single-leg stance phase of gait, hip abductor weakness may lead to pelvic drop and lateral trunk tilt, altering the position of the ground reaction force relative to the knee joint center and thereby affecting the knee adduction moment (Takacs and Hunt, 2012). Abnormal femoral rotation may also alter patellofemoral contact pressure and tibiofemoral shear stress and could contribute to pain and functional impairment in patients with KOA (Duparc et al., 2014) (Figure 3).

**FIGURE 3:**
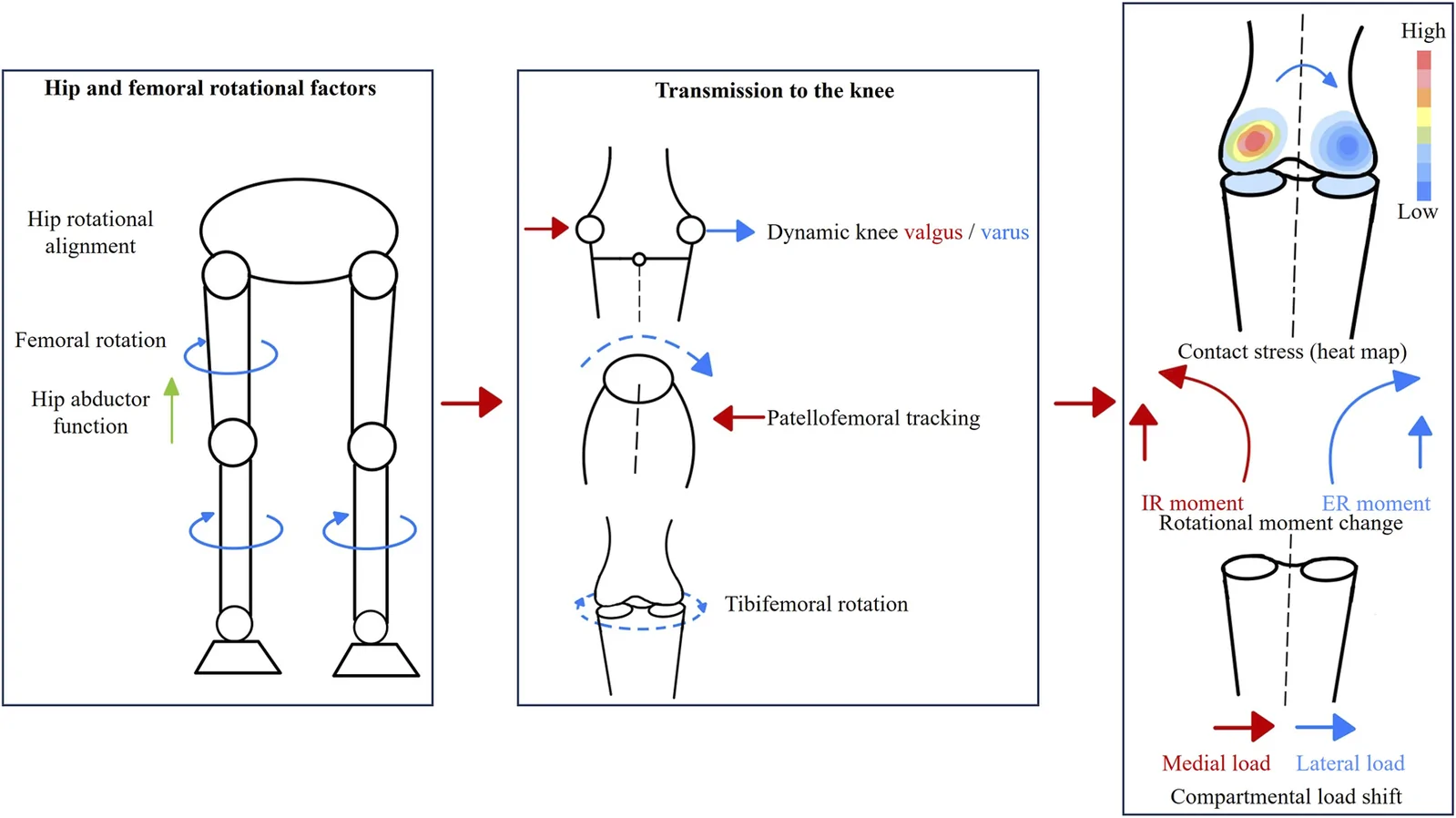
Hip mechanics, femoral rotation, and knee loading.

### 4.3 Ankle-foot posture and knee loading

Limited ankle dorsiflexion range of motion may reduce anterior tibial translation during the stance phase of gait (Malloy et al., 2015; Xian et al., 2025). The body may adopt compensatory strategies, including foot abduction, knee flexion, or alterations in hip movement patterns (Menz et al., 2013; Kruger et al., 2019). Multiplanar abnormalities in foot posture, changes in arch morphology, and altered plantar-pressure distribution may influence tibial rotation and the direction of the ground reaction force, thereby modulating the magnitude of the knee adduction moment and load distribution between the medial and lateral compartments (Nakazato et al., 2025). Changes in foot progression angle may also affect the knee adduction moment by altering the moment arm of the ground reaction force relative to the knee joint center (Rutherford et al., 2008) (Figure 4).

**FIGURE 4:**
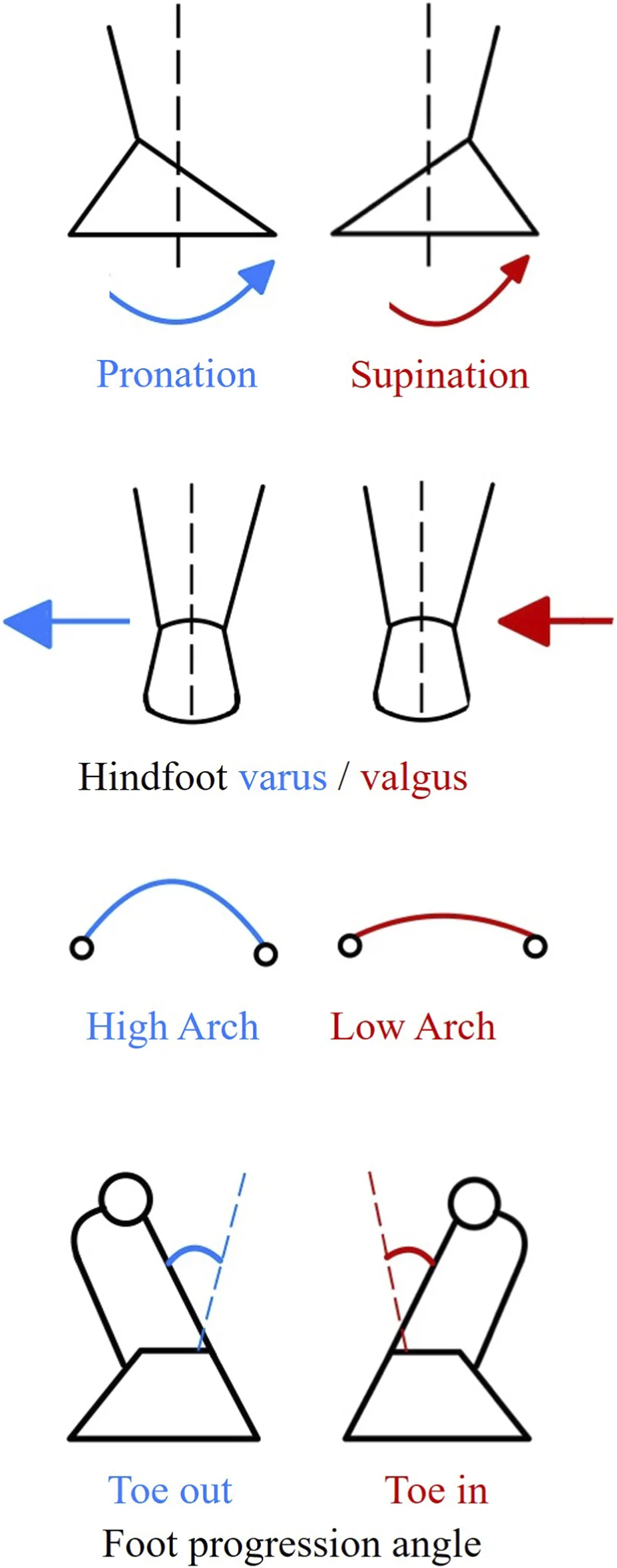
Ankle-foot posture and knee loading.

### 4.4 Malalignment and mechano-inflammation in KOA

Persistent abnormal loading may not only accelerate cartilage damage but also induce inflammatory and catabolic responses in joint tissues through mechanotransduction (Vincent, 2019; Hodgkinson et al., 2022). Chondrocytes can sense abnormal compressive, shear, tensile, and impact loading and translate these physical stimuli into intracellular biochemical signals through mechanosensitive ion channels, extracellular matrix-cytoskeleton linkages, primary cilia, and related transcriptional regulatory networks (Dieterle et al., 2021; Zhang Y. et al., 2023). When mechanical stimulation is excessive, prolonged, or outside the physiological range, it may trigger Ca^2+^ signaling and oxidative stress, activate NF-κB and MAPK pathways and inflammasome signaling, and upregulate the COX-2/PGE2 axis. These changes may promote the expression of inflammatory mediators, pain-related factors, and matrix-degrading enzymes, thereby contributing to a mechano-inflammatory response (Du et al., 2025; Chen et al., 2026; Wan and Zheng, 2026) (Figure 5).

**FIGURE 5:**
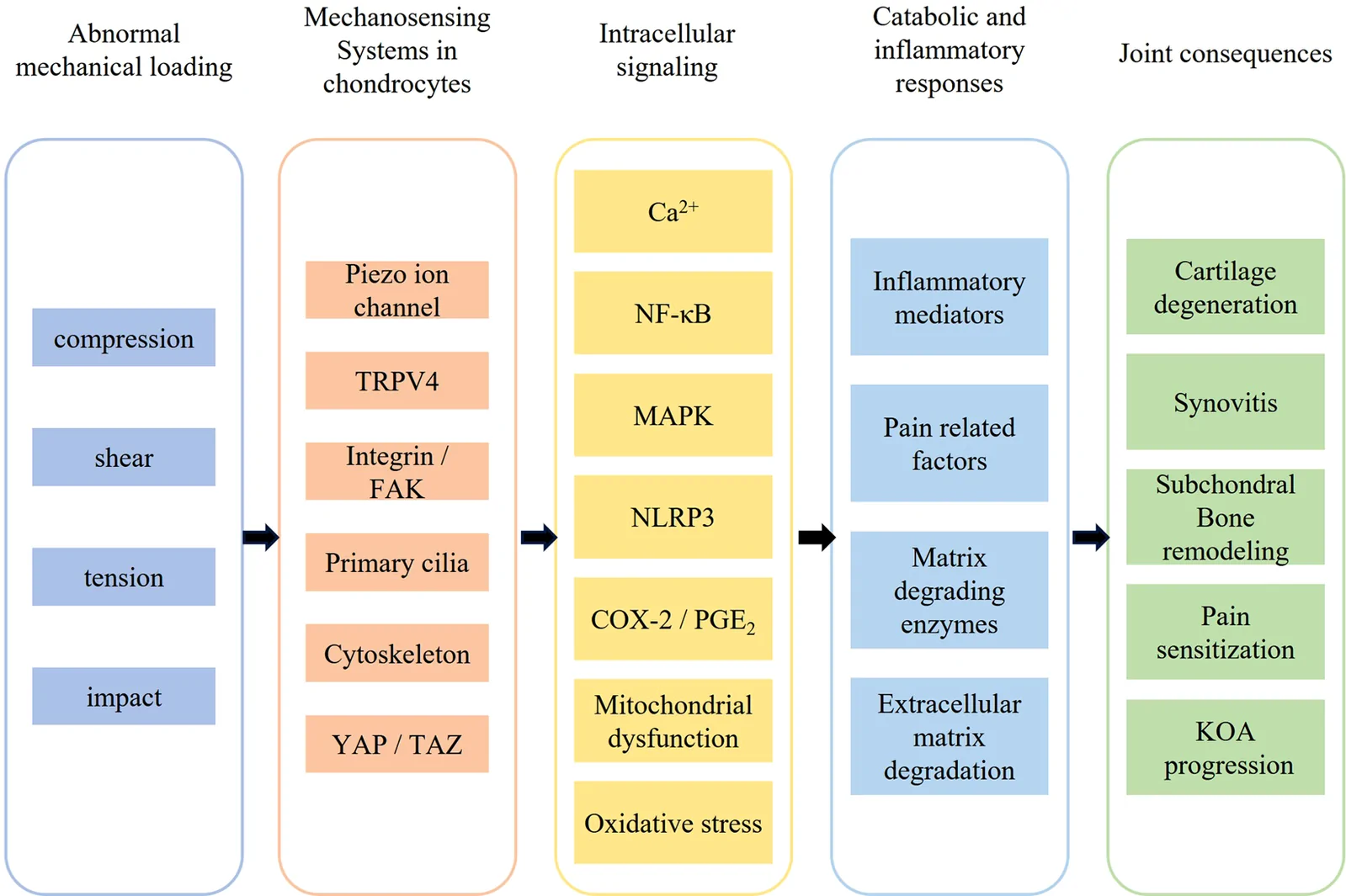
Mechanotransduction and mechano-inflammation.

Abnormal mechanical loading may induce mechano-inflammatory responses in joint tissues, contribute to cartilage matrix degradation, and affect surrounding structures such as the synovium and subchondral bone, thereby shifting chondrocytes from an anabolic toward a catabolic phenotype (Griffin and Guilak, 2005; Felson, 2013). Such inflammatory responses may provide a biological link between global malalignment and local structural deterioration of the knee. When malalignment occurs in the spine, pelvis, or lower extremity, the magnitude, direction, and distribution of loads transmitted to the knee may change (Deveza et al., 2026; Han et al., 2026; Zhang et al., 2026). These abnormal mechanical signals may further promote chondrocyte catabolism, whereas inflammation - associated pain, muscle inhibition, and flexion contracture may in turn worsen postural and gait abnormalities, potentially exacerbating both global and local malalignment (Takahashi et al., 2021; Nakanowatari et al., 2026; Yoon and Moon, 2026).

Global malalignment and local abnormal knee loading may interact and jointly reshape the local mechanical microenvironment in KOA. Abnormal alignment of the spine-pelvis-lower-limb complex may shift the body’s center of mass, alter the transmission pathway of ground reaction forces, and redistribute joint moments across the lower extremity (Kinoshita et al., 2017; Zhang et al., 2021). These changes may lead to load redistribution between the medial and lateral tibiofemoral compartments, shifts in tibiofemoral contact location, and localized tissue stress concentration within the knee (Fu et al., 2023). Conversely, local abnormalities such as varus or valgus deformity, flexion contracture, VT, and pain-protective gait patterns may influence pelvic posture, trunk control, and global compensatory strategies (Katsumi et al., 2023). Together, these reciprocal interactions could create a mechanically imbalanced state in which global and local alignment abnormalities reinforce one another. Consequently, malalignment, altered local loading, mechano-inflammation, pain, and functional impairment may form a self-reinforcing cycle. Within such a cycle, mechano-inflammation may act as a biological amplifier and contribute to cartilage degradation, subchondral bone remodeling, synovitis, pain sensitization, and progressive functional decline (Figure 6). These observations suggest that the pathogenesis of KOA may not be explained by local malalignment alone. Re-examining KOA from the perspective of global-local alignment interactions may provide a more comprehensive understanding of its pathological processes.

**FIGURE 6:**
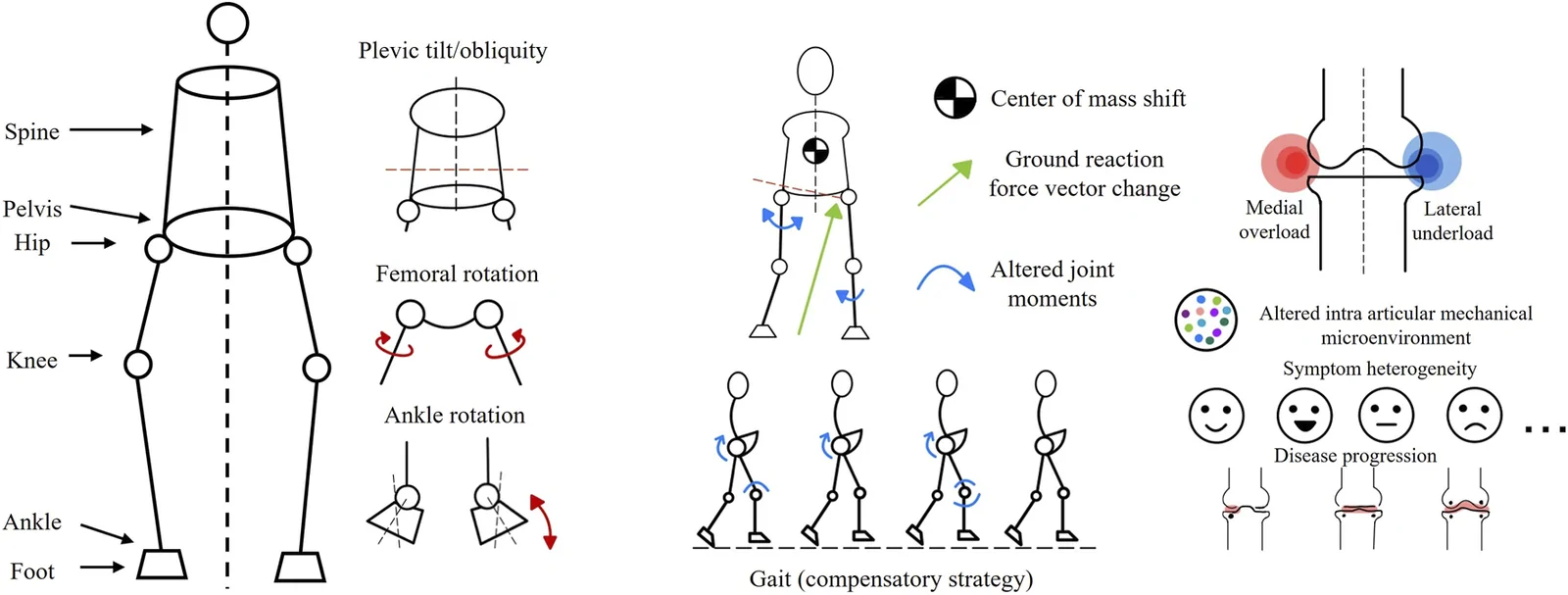
Knee joint loading from a global biomechanical perspective.

### 4.5 Clinical implications and translation

From a clinical perspective, whole chain biomechanical assessment may be incorporated as an extension of conventional evaluation in patients with established KOA. Initial assessment should focus on knee alignment, range of motion, flexion contracture, gait characteristics, and functional limitations (Bach et al., 2002). When compensatory abnormalities are suspected, physical examination can be extended to lumbar spine mobility, pelvic posture, hip range of motion and muscle function, ankle dorsiflexion, foot posture, and bilateral asymmetry (Tan et al., 2020; Park et al., 2022). In selected patients with marked postural abnormalities, complex compensatory patterns, discordance between symptoms and local knee findings, or an inadequate response to conventional treatment, standing radiographic assessment of the spine, pelvis, and lower extremities may provide additional information regarding global alignment.

Integrating functional examination with imaging findings may help identify patient specific abnormalities outside the knee that are potentially associated with altered movement strategies and knee loading. Rather than applying the same whole chain intervention to all patients, treatment could then be directed toward the most relevant and potentially modifiable impairments identified in each individual. However, evidence supporting this approach remains limited, and prospective studies are required to determine whether whole chain assessment improves treatment selection and clinical outcomes compared with conventional knee focused assessment.

Figures 1–6: The preliminary visual concept was developed with the assistance of generative artificial intelligence, and the final figures were manually reconstructed by the authors using Microsoft PowerPoint for Microsoft 365, Version 9, on Windows 11. All graphical elements and labels were manually reviewed and edited for scientific accuracy. No AI-generated image was directly used in the final figures.

## 5 Discussion

This framework suggests that, in addition to evaluating local structural abnormalities and loading changes at the knee, clinical assessment of KOA may benefit from consideration of the spine-pelvis-lower-limb kinetic chain. Alongside conventional local biomechanical parameters, such as the HKA, joint-line obliquity, knee adduction moment, and VT, broader postural and movement-related parameters, including lumbar lordosis, pelvic tilt, trunk posture, hip rotation, ankle-foot alignment, and gait patterns, may also be considered (Nelson et al., 2016; Liao et al., 2019). Such an integrated assessment may allow a more comprehensive identification of the sources of abnormal knee loading and the underlying compensatory mechanisms.

On this basis, stratifying patients according to different biomechanical phenotypes may improve progression-risk assessment and support more individualized intervention. For example, some patients may be characterized primarily by static coronal malalignment, whereas others may present with VT or an increased knee adduction moment (Wink et al., 2017; Kim et al., 2026). In other patients, knee flexion compensation secondary to sagittal spine-pelvis imbalance, or knee load redistribution associated with changes in foot progression angle or ankle-foot posture, may represent the dominant biomechanical feature (Wang et al., 2016; Richards et al., 2018; Hajibabaei et al., 2026; Subasinghe Arachchige and Cheung, 2026). Identifying these distinct biomechanical phenotypes may facilitate a shift from a uniform treatment approach toward phenotype-guided precision management.

For conservative management, interventions such as gait retraining, foot orthoses, knee bracing, strengthening of the periarticular hip and knee muscles, core stability training, and neuromuscular rehabilitation may be selected and combined according to the patient’s predominant mechanical abnormalities (Di Raimondo et al., 2025; Rogoschin et al., 2025; Sabet et al., 2025; Akkaya et al., 2026). For patients with evident compensatory changes across the kinetic chain, intervention targets may extend beyond the knee to adjacent segments, including the spine, pelvis, hip, and ankle-foot complex. Manual therapy, motor-control training, or comprehensive rehabilitation programs may thereby help improve overall load-transmission patterns.

For surgical interventions, such as osteotomy or arthroplasty, preoperative planning and postoperative evaluation may also benefit from consideration of global alignment and compensatory status (Suzuki et al., 2026). If spine-pelvis imbalance, abnormal hip rotation, knee flexion compensation, or persistent ankle-foot postural abnormalities remain unresolved, knee load transmission may not be fully restored even after local coronal alignment correction. Accordingly, combining local alignment correction with efforts to restore global postural balance may help optimize surgical strategies, improve postoperative function, and potentially reduce long-term risks associated with persistent abnormal loading.

In summary, comprehensive biomechanical assessment integrating both local and global alignment, together with stratified intervention strategies based on biomechanical phenotypes, may provide new perspectives for optimizing KOA management, slowing disease progression, and improving long-term functional outcomes.

## 6 Future perspectives

Future research should aim to establish a standardized whole-chain assessment framework that integrates standing full-length radiographs, spinopelvic parameters, three-dimensional gait analysis, wearable sensors, plantar-pressure measurements, and patient-reported outcomes to characterize the biomechanical heterogeneity of KOA. Prospective cohort studies are also needed to clarify the temporal and potentially causal relationships between global malalignment and disease progression.

Moreover, artificial intelligence and wearable technologies, such as inertial sensors, markerless motion capture, smart insoles, and machine learning, may enable continuous monitoring of daily mechanical loading, identification of high-risk gait phenotypes, and guidance of individualized rehabilitation. At the same time, patient-specific biomechanical modeling could be integrated with tissue-imaging biomarkers, synovial fluid analysis, and multi-omics approaches to clarify the mechanisms by which abnormal external mechanical loading may be translated into intra-articular inflammation and degeneration. Ultimately, randomized controlled trials will be needed to evaluate the efficacy of whole-chain biomechanical correction and to identify optimal combinations of interventions and objective biomechanical endpoints.

The current evidence base is limited by substantial heterogeneity, the predominance of cross-sectional and association-based study designs, insufficient standardization and validation of internal biomechanical models, and the reliance of mechanobiological evidence mainly on animal and cellular experiments. Consequently, causal relationships remain uncertain, and the clinical relevance of many mechanobiological findings requires further confirmation. In addition, the proposed concept that global malalignment may aggravate local loading and subsequently promote mechanically mediated inflammatory progression requires further validation across distinct biomechanical phenotypes.

## 7 Conclusion

Knee osteoarthritis should not be viewed solely as a localized disorder of the knee but may also involve complex interactions between abnormal mechanical loading and inflammatory responses. Local loading abnormalities may interact with malalignment and compensatory changes across the spine-pelvis-lower-limb kinetic chain, while mechano-inflammation may amplify tissue degeneration and contribute to pain and functional impairment. An integrated perspective that considers both whole-chain biomechanics and local mechano-inflammatory responses may help improve biomechanical phenotyping, risk stratification, and individualized interventions. However, this framework remains largely conceptual and requires further validation in prospective cohort studies and well-designed interventional trials.

## 8 Limitation

Several limitations of this narrative review should be acknowledged. First, although the review integrates evidence concerning global alignment, local knee loading, and mechanobiological responses, some potentially important components of the osteochondral mechanical environment were not examined in detail. In particular, the distinct microarchitecture and mechanical properties of the subchondral cortical plate and trabecular bone were beyond the primary scope of this review. These structures may respond differently to abnormal loading and may in turn influence local load transmission and stress distribution. Second, the proposed relationships among knee malalignment, subchondral bone remodeling, and compensatory loading across the kinetic chain are likely bidirectional and multifactorial. However, direct longitudinal and experimental evidence linking these processes remains limited. Third, the evidence summarized in this review was derived from heterogeneous human, animal, *in vitro*, and modeling studies, which differ substantially in their biological scale and clinical generalizability. Therefore, several mechanistic relationships discussed in this review should be interpreted as biologically plausible rather than definitively causal. Finally, standardized thresholds for clinically meaningful whole chain malalignment have not been established, and prospective studies are required to determine whether whole chain assessment improves biomechanical phenotyping, treatment selection, or clinical outcomes in KOA.

## References

[B1] Akkaya Z. Joseph G. B. Ziegeler K. Sims W. M. Lynch J. A. Kreutzinger V. (2026). Ultra-processed foods and muscle fat infiltration at thigh MRI: data from the osteoarthritis initiative. Radiology 319, e251129. 10.1148/radiol.251129 PMC13139946 41979462

[B2] Ambrus M. Wolf G. Molnár D. Soussi B. Horváth T. Horváth M. (2026). Effectiveness of pelvic stabilization exercises on knee valgus, muscle activity, and strength in individuals with dynamic knee valgus. BMC Musculoskelet. Disord. 27, 163. 10.1186/s12891-026-09556-9 PMC12924297 41606722

[B3] Asay J. L. Erhart-Hledik J. C. Andriacchi T. P. (2018). Changes in the total knee joint moment in patients with medial compartment knee osteoarthritis over 5 years. J. Orthop. Res. Off. Publ. Orthop. Res. Soc. 36, 2373–2379. 10.1002/jor.23908 29611879

[B4] Bach C. M. Nogler M. Steingruber I. E. Ogon M. Wimmer C. Göbel G. (2002). Scoring systems in total knee arthroplasty. Clin. Orthop. 399 184–196. 10.1097/00003086-200206000-00022 12011708

[B5] Bacon K. L. Felson D. T. Jafarzadeh S. R. Kolachalama V. B. Hausdorff J. M. Gazit E. (2024). Gait alterations and association with worsening knee pain and physical function: a machine learning approach with wearable sensors in the multicenter osteoarthritis study. Arthritis Care Res. 76, 984–992. 10.1002/acr.25327 PMC11209802 38523250

[B6] Baghi R. Yin W. Badhyal S. Ramadan A. Oppizzi G. Li Z. (2025). Determining the individual relationship between the step width and peak knee adduction moment during stepping in medial knee osteoarthritis. Clin. Biomech. Bristol Avon 128, 106619. 10.1016/j.clinbiomech.2025.106619 PMC12368841 40737736

[B7] Balmaceno-Criss M. Lafage R. Alsoof D. Daher M. Hamilton D. K. Smith J. S. (2024). Impact of hip and knee osteoarthritis on full body sagittal alignment and compensation for sagittal spinal deformity. Spine 49, 743–751. 10.1097/BRS.0000000000004957 38375611

[B8] Baniasad M. Martin R. Crevoisier X. Pichonnaz C. Becce F. Aminian K. (2022). Knee adduction moment decomposition: toward better clinical decision-making. Front. Bioeng. Biotechnol. 10, 1017711. 10.3389/fbioe.2022.1017711 PMC9715598 36466350

[B9] Batarfi W. A. Yunus M. H. M. Hamid A. A. Maarof M. Abdul Rani R. (2025). Breaking down osteoarthritis: exploring inflammatory and mechanical signaling pathways. Life 15, 1238. 10.3390/life15081238 PMC12387175 40868886

[B10] Bedson J. Croft P. R. (2008). The discordance between clinical and radiographic knee osteoarthritis: a systematic search and summary of the literature. BMC Musculoskelet. Disord. 9, 116. 10.1186/1471-2474-9-116 18764949 PMC2542996

[B11] Ben Hassine S. Balti A. Abid S. Ben Khelifa M. M. Sayadi M. (2024). Markerless vision-based knee osteoarthritis classification using machine learning and gait videos. Front. Signal Process. 4, 1479244. 10.3389/frsip.2024.1479244

[B12] Bennell K. L. Bowles K.-A. Wang Y. Cicuttini F. Davies-Tuck M. Hinman R. S. (2011). Higher dynamic medial knee load predicts greater cartilage loss over 12 months in medial knee osteoarthritis. Ann. Rheum. Dis. 70, 1770–1774. 10.1136/ard.2010.147082 21742637

[B13] Bg H. S E. We M. Pl S. (2025). The discordance between pain and imaging in knee osteoarthritis. J. Am. Acad. Orthop. Surg. 33. (14), e786–e794. 10.5435/JAAOS-D-24-00509 39965186

[B14] Brylka L. J. Alimy A.-R. Tschaffon-Müller M. E. A. Jiang S. Ballhause T. M. Baranowsky A. (2024). Piezo1 expression in chondrocytes controls endochondral ossification and osteoarthritis development. Bone Res. 12, 12. 10.1038/s41413-024-00315-x 38395992 PMC10891122

[B15] Cappozzo A. Della Croce U. Leardini A. Chiari L. (2005). Human movement analysis using stereophotogrammetry. Part 1: theoretical background. Gait Posture 21, 186–196. 10.1016/j.gaitpost.2004.01.010 15639398

[B16] Chang A. Hochberg M. Song J. Dunlop D. Chmiel J. S. Nevitt M. (2010). Frequency of varus and valgus thrust and factors associated with thrust presence in persons with or at higher risk of developing knee osteoarthritis. Arthritis Rheum. 62, 1403–1411. 10.1002/art.27377 20213800 PMC2921866

[B17] Chang A. H. Chmiel J. S. Moisio K. C. Almagor O. Zhang Y. Cahue S. (2013). Varus thrust and knee frontal plane dynamic motion in persons with knee osteoarthritis. Osteoarthr. Cartil. 21, 1668–1673. 10.1016/j.joca.2013.08.007 23948980 PMC4014355

[B18] Chen S. Li Z. Zhang J. Liu H. (2026). Piezo1 at the crossroads: mediating inflammation and mechanical stress in joint disorders. Jt. Bone Spine 93, 105953. 10.1016/j.jbspin.2025.105953 40738358

[B19] Cho Y. Jo D. J. Hyun S.-J. Park J. H. Yang N. R. (2023). From the spinopelvic parameters to global alignment and proportion scores in adult spinal deformity. Neurospine 20, 467–477. 10.14245/ns.2346374.187 37401065 PMC10323335

[B20] Corrigan P. Felson D. T. Lewis C. L. Neogi T. LaValley M. P. Gross K. D. (2023). Relation of temporal asymmetry during walking to two-year knee pain outcomes in those with mild-to-moderate unilateral knee pain: an exploratory analysis from the multicenter osteoarthritis study. Arthritis Care Res. 75, 1735–1743. 10.1002/acr.25050 PMC10133409 36305013

[B21] Deveza L. Ani F. Perrier G. Maglaras C. O’Connell B. Raman T. (2026). The impact of knee flexion on global alignment in spinal deformity: a radiographic study. Clin. Spine Surg. 10.1097/BSD.0000000000001932 41926444

[B22] Di Bacco V. E. Masood Z. Keogh J. A. J. Ruder M. C. Gafoor F. Wu J. (2025). Defining and measuring varus thrust in knee osteoarthritis: a scoping review of current evidence and challenges. Osteoarthr. Cartil. Open 7, 100683. 10.1016/j.ocarto.2025.100683 41050369 PMC12495470

[B23] Di Raimondo G. Willems M. Killen B. A. Havashinezhadian S. Turcot K. Vanwanseele B. (2025). The impact of PCA derived gait kinematic variations on estimated medial knee contact forces in a knee osteoarthritis population. Sci. Rep. 15, 18342. 10.1038/s41598-025-90804-8 PMC12106812 40419706

[B24] Dieterle M. P. Husari A. Rolauffs B. Steinberg T. Tomakidi P. (2021). Integrins, cadherins and channels in cartilage mechanotransduction: perspectives for future regeneration strategies. Expert Rev. Mol. Med. 23, e14. 10.1017/erm.2021.16 34702419 PMC8724267

[B25] Du P. Yan Y. Yin S. Li X. Wang H. Sun J. (2024). Correlation between coronal position sequence of lumbar and WOMAC score in knee osteoarthritis (KOA) in standard standing position. Adv. Ther. 41, 2924–2935. 10.1007/s12325-024-02898-7 38833141

[B26] Du X. Liu R. Jiang Z. Zhang C. Yang Z. Hu S. (2025). Chondrocyte lysates activate NLRP3 inflammasome-induced pyroptosis in synovial fibroblasts to exacerbate knee synovitis by downregulating caveolin-1. Arthritis Res. Ther. 27, 104. 10.1186/s13075-025-03573-0 40375346 PMC12083164

[B27] Duparc F. Thomine J. M. Simonet J. Biga N. (2014). Femoral and tibial bone torsions associated with medial femoro-tibial osteoarthritis. Index of cumulative torsions. Orthop. Traumatol. Surg. Res. 100, 69–74. 10.1016/j.otsr.2013.12.014 24456764

[B28] Felson D. T. (2013). Osteoarthritis as a disease of mechanics. Osteoarthr. Cartil. 21, 10–15. 10.1016/j.joca.2012.09.012 23041436 PMC3538894

[B29] Felson D. T. Niu J. Neogi T. Goggins J. Nevitt M. C. Roemer F. (2016). Synovitis and the risk of knee osteoarthritis: the MOST study. Osteoarthr. Cartil. 24, 458–464. 10.1016/j.joca.2015.09.013 PMC4761323 26432512

[B30] Fu P. Xu W. Xu P. Huang J. Guo J. J. (2023). Relationship between spinal imbalance and knee osteoarthritis by using full-body EOS. BMC Musculoskelet. Disord. 24, 402. 10.1186/s12891-023-06508-5 37208648 PMC10197256

[B31] GBD 2021 Osteoarthritis Collaborators, Culbreth G. T. Haile L. M. Rafferty Q. Lo J. Fukutaki K. G. (2023). Global, regional, and national burden of osteoarthritis, 1990-2020 and projections to 2050: a systematic analysis for the global burden of disease study 2021. Lancet Rheumatol. 5, e508–e522. 10.1016/S2665-9913(23)00163-7 37675071 PMC10477960

[B32] Griffin T. M. Guilak F. (2005). The role of mechanical loading in the onset and progression of osteoarthritis. Exerc. Sport Sci. Rev. 33, 195–200. 10.1097/00003677-200510000-00008 16239837

[B33] Gustafson J. A. Anderton W. Sowa G. A. Piva S. R. Farrokhi S. (2019). Dynamic knee joint stiffness and contralateral knee joint loading during prolonged walking in patients with unilateral knee osteoarthritis. Gait Posture 68, 44–49. 10.1016/j.gaitpost.2018.10.032 30453143 PMC6370490

[B34] Hajibabaei F. Daryabor A. Arazpour M. Biglariyan A. Abolghasemi S. (2026). Effect of an ankle-foot orthosis on gait parameters in medial knee osteoarthritis: a single-subject study. Assist. Technol., 1–8. 10.1080/10400435.2026.2659185 42262334

[B35] Han Y. Jeon S. Woo H. Ha W. Park T.-Y. Lee J.-H. (2026). Association between calcaneal inclination angle and spinal and lower limb alignment: a retrospective radiographic analysis. Diagnostics 16, 921. 10.3390/diagnostics16060921 PMC13024928 41897654

[B36] Hodgkinson T. Kelly D. C. Curtin C. M. O’Brien F. J. (2022). Mechanosignalling in cartilage: an emerging target for the treatment of osteoarthritis. Nat. Rev. Rheumatol. 18, 67–84. 10.1038/s41584-021-00724-w 34934171

[B37] Iijima H. Shimoura K. Ono T. Aoyama T. Takahashi M. (2019). Proximal gait adaptations in individuals with knee osteoarthritis: a systematic review and meta-analysis. J. Biomech. 87, 127–141. 10.1016/j.jbiomech.2019.02.027 30904334

[B38] Jahn J. Ehlen Q. T. Huang C.-Y. (2024). Finding the goldilocks zone of mechanical loading: a comprehensive review of mechanical loading in the prevention and treatment of knee osteoarthritis. Bioengineering 11, 110. 10.3390/bioengineering11020110 PMC10886318 38391596

[B39] Jia Y. Le H. Wang X. Zhang J. Liu Y. Ding J. (2023). Double-edged role of mechanical stimuli and underlying mechanisms in cartilage tissue engineering. Front. Bioeng. Biotechnol. 11, 1271762. 10.3389/fbioe.2023.1271762 PMC10694366 38053849

[B40] Kanthawang T. Bodden J. Joseph G. B. Lane N. E. Nevitt M. McCulloch C. E. (2021). Obese and overweight individuals have greater knee synovial inflammation and associated structural and cartilage compositional degeneration: data from the osteoarthritis initiative. Skelet. Radiol. 50, 217–229. 10.1007/s00256-020-03550-5 32699956 PMC7677197

[B41] Katsumi R. Mannen E. M. Bajaj G. Smith J. R. Mears S. C. Stambough J. B. (2023). The influence of knee osteoarthritis on spinopelvic alignment and global sagittal balance. J. Knee Surg. 36, 917–924. 10.1055/s-0042-1747947 35605970

[B42] Kiernan D. Katzman Z. D. Hawkins D. A. Christiansen B. A. (2024). A 0.05 m change in inertial measurement unit placement alters time and frequency domain metrics during running. Sensors 24, 656. 10.3390/s24020656 38276348 PMC10820910

[B43] Kim J.-S. MacDessi S. J. Lee D. W. Ro D. H. (2026). Coronal alignment parameters of the knee predict osteoarthritis development: a coronal plane alignment of the knee classification-based analysis using the multicenter osteoarthritis study data. Bone Jt. J. 108-B, 889–897. 10.1302/0301-620X.108B7.BJJ-2025-0687.R2 42379578

[B44] Kinoshita K. Ishida K. Hashimoto M. Yoneda Y. Naka Y. Kitanishi H. (2017). Relationship between trunk function evaluated using the trunk righting test and physical function in patients with knee osteoarthritis. J. Phys. Ther. Sci. 29, 996–1000. 10.1589/jpts.29.996 28626308 PMC5468223

[B45] Knebel A. Daher M. Singh M. Fisher L. Daniels A. H. Diebo B. G. (2024). Sagittal spinal alignment measurements and evaluation: historical perspective. North Am. Spine Soc. J. 19, 100519. 10.1016/j.xnsj.2024.100519 PMC11345912 39188671

[B46] Kobsar D. Charlton J. M. Tse C. T. F. Esculier J.-F. Graffos A. Krowchuk N. M. (2020). Validity and reliability of wearable inertial sensors in healthy adult walking: a systematic review and meta-analysis. J. Neuro Engineering Rehabil. 17, 62. 10.1186/s12984-020-00685-3 32393301 PMC7216606

[B47] Kruger K. M. Graf A. Flanagan A. McHenry B. D. Altiok H. Smith P. A. (2019). Segmental foot and ankle kinematic differences between rectus, planus, and cavus foot types. J. Biomech. 94, 180–186. 10.1016/j.jbiomech.2019.07.032 31420153

[B48] Kutzner I. Trepczynski A. Heller M. O. Bergmann G. (2013). Knee adduction moment and medial contact force – facts about their correlation during gait. PLoS One 8, e81036. 10.1371/journal.pone.0081036 24312522 PMC3847086

[B49] Lafage R. Duvvuri P. Elysee J. Diebo B. Bess S. Burton D. (2023). Quantifying the contribution of lower limb compensation to upright posture: what happens if adult spinal deformity patients do not compensate? Spine 48, 1082–1088. 10.1097/BRS.0000000000004646 36972137

[B50] Li G. Li S. Xie J. Zhang Z. Zou J. Yang C. (2024). Identifying changes in dynamic plantar pressure associated with radiological knee osteoarthritis based on machine learning and wearable devices. J. Neuroeng. Rehabil. 21, 45. 10.1186/s12984-024-01337-6 38570841 PMC10988837

[B51] Liao T.-C. Samaan M. A. Popovic T. Neumann J. Zhang A. L. Link T. M. (2019). Abnormal joint loading during gait in persons with hip osteoarthritis is associated with symptoms and cartilage lesions. J. Orthop. Sports Phys. Ther. 49, 917–924. 10.2519/jospt.2019.8945 31610757 PMC7935417

[B52] Liu S. Amiri P. McGregor A. H. Bull A. M. J. (2023). Bilateral asymmetry in knee and hip musculoskeletal loading during stair ascending/descending in individuals with unilateral mild-to-moderate medial knee osteoarthritis. Ann. Biomed. Eng. 51, 2490–2503. 10.1007/s10439-023-03289-9 37482575 PMC10598163

[B53] Liu Y. Wang Y. Yan P. Cui N. Xu K. Liu D. (2025). NLRP3 inflammasome-mediated osteoarthritis: the role of epigenetics. Biology 14, 71. 10.3390/biology14010071 PMC11761621 39857301

[B54] Malloy P. Morgan A. Meinerz C. Geiser C. Kipp K. (2015). The association of dorsiflexion flexibility on knee kinematics and kinetics during a drop vertical jump in healthy female athletes. Knee Surg. Sports Traumatol. Arthrosc. 23, 3550–3555. 10.1007/s00167-014-3222-z 25112598 PMC4977993

[B55] Manal K. Gardinier E. Buchanan T. S. Snyder-Mackler L. (2015). A more informed evaluation of medial compartment loading: the combined use of the knee adduction and flexor moments. Osteoarthr. Cartil. 23, 1107–1111. 10.1016/j.joca.2015.02.779 25862486 PMC4470852

[B56] Marques Luís N. Varatojo R. (2021). Radiological assessment of lower limb alignment. EFORT Open Rev. 6, 487–494. 10.1302/2058-5241.6.210015 34267938 PMC8246117

[B57] Matsuda T. Watanabe J. Sotokawa T. Shishime T. Katoh H. (2025). Estimation of the external knee adduction moment using inertial measurement unit sensors on the shank and lower back: a pilot study. J. Funct. Morphol. Kinesiol. 10, 356. 10.3390/jfmk10030356 40981055 PMC12452454

[B58] Menz H. B. Dufour A. B. Riskowski J. L. Hillstrom H. J. Hannan M. T. (2013). Foot posture, foot function and low back pain: the Framingham foot study. Rheumatology 52, 2275–2282. 10.1093/rheumatology/ket298 24049103 PMC3828513

[B59] Michalska-Foryszewska A. Modzelewski P. Sklinda K. Mruk B. Walecki J. (2024). Radiological approach to assessment of lower-limb alignment—coronal and transverse plane analysis. J. Clin. Med. 13, 6975. 10.3390/jcm13226975 39598119 PMC11595539

[B60] Micó-Amigo M. E. Bonci T. Paraschiv-Ionescu A. Ullrich M. Kirk C. Soltani A. (2023). Assessing real-world gait with digital technology? Validation, insights and recommendations from the Mobilise-D consortium. J. Neuro Eng. Rehabil. 20, 78. 10.1186/s12984-023-01198-5 PMC10265910 37316858

[B61] Misu S. Tanaka S. Ishihara K. Asai T. Nishigami T. (2022). Applied assessment method for varus thrust during walking in patients with knee osteoarthritis using acceleration data measured by an inertial measurement unit. Sensors 22, 6460. 10.3390/s22176460 36080919 PMC9460931

[B62] Misu S. Tanaka S. Miura J. Ishihara K. Asai T. Nishigami T. (2023). Association of the degree of varus thrust during gait assessed by an inertial measurement unit with patient-reported outcome measures in knee osteoarthritis. Sensors 23, 4578. 10.3390/s23104578 37430491 PMC10224415

[B63] Miyazaki T. Wada M. Kawahara H. Sato M. Baba H. Shimada S. (2002). Dynamic load at baseline can predict radiographic disease progression in medial compartment knee osteoarthritis. Ann. Rheum. Dis. 61, 617–622. 10.1136/ard.61.7.617 12079903 PMC1754164

[B64] Mononen M. E. Paz A. Liukkonen M. K. Turunen M. J. (2023). Atlas-based finite element analyses with simpler constitutive models predict personalized progression of knee osteoarthritis: data from the osteoarthritis initiative. Sci. Rep. 13, 8888. 10.1038/s41598-023-35832-y 37264050 PMC10235100

[B65] Moura F. A. Pelegrinelli A. R. M. Catelli D. S. Kowalski E. Lamontagne M. da Silva Torres R. (2024). On the prediction of tibiofemoral contact forces for healthy individuals and osteoarthritis patients during gait: a comparative study of regression methods. Sci. Rep. 14, 1379. 10.1038/s41598-023-50481-x 38228640 PMC10791669

[B66] Mündermann A. Nüesch C. Ewald H. Jonkers I. (2024). Osteoarthritis year in review 2024: biomechanics. Osteoarthr. Cartil. 32, 1530–1541. 10.1016/j.joca.2024.09.011 39369839

[B67] Murakami Y. Hanada M. Nomoto K. Hotta K. Yamagishi Y. Matsuyama Y. (2025). Body mass index and spinopelvic alignment as predictors of incident knee osteoarthritis: an 8-Year longitudinal study from the TOEI cohort of older Japanese women. J. Clin. Med. 14, 7343. 10.3390/jcm14207343 41156213 PMC12565032

[B68] Nakanowatari T. Akiyama C. Koho T. Kobayashi A. Kato Y. Hoshi M. (2026). Asymmetric plantar loading during gait is prospectively associated with early changes in knee and hip pain in community-dwelling older adults. Clin. Biomech. 139, 106939. 10.1016/j.clinbiomech.2026.106939 42551219

[B69] Nakazato K. Pataky T. Taniguchi M. Saeki J. Yagi M. Motomura Y. (2025). Center of pressure in relation to foot morphology and knee symptoms in older women with medial knee joint deformity: a cross-sectional study. Arch. Phys. Med. Rehabil. 106, 887–893. 10.1016/j.apmr.2024.10.015 39549825

[B70] Nelson A. E. Golightly Y. M. Renner J. B. Schwartz T. A. Liu F. Lynch J. A. (2016). Variations in hip shape are associated with radiographic knee osteoarthritis: cross-sectional and longitudinal analyses of the Johnston county osteoarthritis project. J. Rheumatol. 43, 405–410. 10.3899/jrheum.150559 26669914 PMC4738023

[B71] Nims R. Palmer D. R. Kassab J. Zhang B. Guilak F. (2024). The chondrocyte “mechanome”: activation of the mechanosensitive ion channels TRPV4 and PIEZO1 drives unique transcriptional signatures. FASEB J. 38, e23778. 10.1096/fj.202400883R PMC11327906 38959010

[B72] Nishino K. Omori G. Koga Y. Kobayashi K. Sakamoto M. Tanabe Y. (2015). Three-dimensional dynamic analysis of knee joint during gait in medial knee osteoarthritis using loading axis of knee. Gait Posture 42, 127–132. 10.1016/j.gaitpost.2015.04.018 26002602

[B73] Park J. Y. Cho B. W. Kwon H. M. Park K. K. Lee W.-S. (2022). Knee extension is related to the posteriorly deviated gravity line to the pelvis in young adults: radiographic analysis using low-dose biplanar X-ray. Yonsei Med. J. 63, 933–940. 10.3349/ymj.2022.0184 PMC9520046 36168246

[B74] Paterson K. L. Hinman R. S. Metcalf B. R. Bennell K. L. Wrigley T. V. (2017). Plug-in-Gait calculation of the knee adduction moment in people with knee osteoarthritis during shod walking: comparison of two different foot marker models. J. Foot Ankle Res. 10, 8. 10.1186/s13047-017-0187-4 28174605 PMC5292150

[B75] Poole A. R. (2012). Osteoarthritis as a whole joint disease. HSS J. Musculoskelet. J. Hosp. Spec. Surg. 8, 4–6. 10.1007/s11420-011-9248-6 23372516 PMC3295952

[B76] Pratobevera A. Seil R. Menetrey J. (2024). Joint line and knee osteotomy. EFORT Open Rev. 9, 375–386. 10.1530/EOR-24-0037 38726996 PMC11099584

[B77] Richards R. van der Esch M. van den Noort J. C. Harlaar J. (2018). The learning process of gait retraining using real-time feedback in patients with medial knee osteoarthritis. Gait Posture 62, 1–6. 10.1016/j.gaitpost.2018.02.023 29494998

[B78] Roemer F. W. Jarraya M. Collins J. E. Kwoh C. K. Hayashi D. Hunter D. J. (2023). Structural phenotypes of knee osteoarthritis: potential clinical and research relevance. Skelet. Radiol. 52, 2021–2030. 10.1007/s00256-022-04191-6 PMC10509066 36161341

[B79] Rogoschin J. Preiser T. Hansen L. Potthast W. Komnik I. (2025). Kinetic effects of unicompartmental unloader braces in medial knee osteoarthritis: a longitudinal study of joint load during level, stair, and slope walking. J. Biomech. 184, 112593. 10.1016/j.jbiomech.2025.112593 40139121

[B80] Rutherford D. J. Hubley-Kozey C. L. Deluzio K. J. Stanish W. D. Dunbar M. (2008). Foot progression angle and the knee adduction moment: a cross-sectional investigation in knee osteoarthritis. Osteoarthr. Cartil. 16, 883–889. 10.1016/j.joca.2007.11.012 18182310

[B81] Sabet F. Anbarian M. Kainz H. Gonçalves B. (2025). Acute effects of lateral wedge insoles on lower limb joint kinematics and symptoms in women with medial compartment knee osteoarthritis during walking. Gait Posture 122, 272–278. 10.1016/j.gaitpost.2025.07.319 40768789

[B82] Sanchez-Adams J. Leddy H. A. McNulty A. L. O’Conor C. J. Guilak F. (2014). The mechanobiology of articular cartilage: bearing the burden of osteoarthritis. Curr. Rheumatol. Rep. 16, 451. 10.1007/s11926-014-0451-6 25182679 PMC4682660

[B83] Sharma L. (2021). Osteoarthritis of the knee. N. Engl. J. Med. 384, 51–59. 10.1056/NEJMcp1903768 33406330

[B84] Sharma L. Song J. Felson D. T. Cahue S. Shamiyeh E. Dunlop D. D. (2001). The role of knee alignment in disease progression and functional decline in knee osteoarthritis. JAMA 286, 188–195. 10.1001/jama.286.2.188 11448282

[B85] Sharma L. Song J. Dunlop D. Felson D. Lewis C. E. Segal N. (2010). Varus and valgus alignment and incident and progressive knee osteoarthritis. Ann. Rheum. Dis. 69, 1940–1945. 10.1136/ard.2010.129742 20511608 PMC2994600

[B86] Sharma L. Chang A. H. Jackson R. D. Nevitt M. Moisio K. C. Hochberg M. (2017). Varus thrust and incident and progressive knee osteoarthritis. Arthritis Rheumatol. 69, 2136–2143. 10.1002/art.40224 28772066 PMC5659924

[B87] Singh R. Yadav P. Agarwal S. Kaur S. Jain M. (2025). Changes in spino-pelvis-lower extremity alignment in patients with knee osteoarthritis: a prospective radiographic study. Musculoskelet. Surg. 109, 429–443. 10.1007/s12306-025-00889-7 39921806

[B88] Snyder S. J. Chu E. Um J. Heo Y. J. Miller R. H. Shim J. K. (2023). Prediction of knee adduction moment using innovative instrumented insole and deep learning neural networks in healthy female individuals. Knee 41, 115–123. 10.1016/j.knee.2022.12.007 36657209

[B89] Subasinghe Arachchige R. S. S. Cheung R. T. H. (2026). Relationship between knee adduction moment and knee contact forces during walking and running with modified foot progression angles. J. Biomech. 205, 113443. 10.1016/j.jbiomech.2026.113443 42385311

[B90] Suzuki Y. Ohkoshi Y. Kawakami K. Shimizu K. Chida S. Ukishiro K. (2023). Assessing knee joint biomechanics and trunk posture according to medial osteoarthritis severity. Sci. Rep. 13, 19186. 10.1038/s41598-023-46486-1 37932370 PMC10628121

[B91] Suzuki Y. Sakurai S. Kawakami K. Ukishiro K. Onodera T. Iwasaki K. (2026). Effects of medial opening-wedge high tibial osteotomy on 3-Dimensional knee joint biomechanics and trunk posture. Orthop. J. Sports Med. 14, 23259671261450211. 10.1177/23259671261450211 PMC13328983 42404927

[B92] Takacs J. Hunt M. A. (2012). The effect of contralateral pelvic drop and trunk lean on frontal plane knee biomechanics during single limb standing. J. Biomech. 45, 2791–2796. 10.1016/j.jbiomech.2012.08.041 22999376

[B93] Takahashi S. Hoshino M. Ohyama S. Hori Y. Yabu A. Kobayashi A. (2021). Relationship of back muscle and knee extensors with the compensatory mechanism of sagittal alignment in a community-dwelling elderly population. Sci. Rep. 11, 2179. 10.1038/s41598-021-82015-8 33500554 PMC7838190

[B94] Tan J. M. Crossley K. M. Munteanu S. E. Collins N. J. Hart H. F. Donnar J. W. (2020). Associations of foot and ankle characteristics with knee symptoms and function in individuals with patellofemoral osteoarthritis. J. Foot Ankle Res. 13, 57. 10.1186/s13047-020-00426-8 32967701 PMC7509922

[B95] Thomas D. T. R S. Prabhakar A. J. Dineshbhai P. V. Eapen C. (2022). Hip abductor strengthening in patients diagnosed with knee osteoarthritis – a systematic review and meta-analysis. BMC Musculoskelet. Disord. 23, 622. 10.1186/s12891-022-05557-6 35768802 PMC9241212

[B96] Tomay Aksoy Ö. Bütün B. (2025). The relationship between spinopelvic alignment and knee osteoarthritis in female patients: a cross-sectional study. J. Back Musculoskelet. Rehabil. 38, 995–1003. 10.1177/10538127251321767 40101274

[B97] Uhlrich S. D. Kolesar J. A. Kidziński Ł. Boswell M. A. Silder A. Gold G. E. (2022). Personalization improves the biomechanical efficacy of foot progression angle modifications in individuals with medial knee osteoarthritis. J. Biomech. 144, 111312. 10.1016/j.jbiomech.2022.111312 36191434 PMC9889103

[B98] Uhlrich S. D. Mazzoli V. Silder A. Finlay A. K. Kogan F. Gold G. E. (2025). Personalised gait retraining for medial compartment knee osteoarthritis: a randomised controlled trial. Lancet Rheumatol. 7, e708–e718. 10.1016/S2665-9913(25)00151-1 40816302 PMC13034617

[B99] Ulrich B. Erhart-Hledik J. C. Asay J. L. Omoumi P. Andriacchi T. P. Jolles B. M. (2023). Diverse parameters of ambulatory knee moments differ with medial knee osteoarthritis severity and are combinable into a severity index. Front. Bioeng. Biotechnol. 11, 1176471. 10.3389/fbioe.2023.1176471 37383522 PMC10293674

[B100] Vincent T. L. (2019). Mechanoflammation in osteoarthritis pathogenesis. Semin. Arthritis Rheum. 49, S36–S38. 10.1016/j.semarthrit.2019.09.018 31779850

[B101] Walter J. P. D’Lima D. D. Colwell C. W. Fregly B. J. (2010). Decreased knee adduction moment does not guarantee decreased medial contact force during gait. J. Orthop. Res. 28, 1348–1354. 10.1002/jor.21142 PMC2984615 20839320

[B102] Wan P. Zheng Y. (2026). NF-κB signaling in osteoarthritis: integrating mechanical stress, innate immunity, and cartilage degeneration. Front. Immunol. 17, 1842443. 10.3389/fimmu.2026.1842443 42327716 PMC13278980

[B103] Wang W. J. Liu F. Zhu Y. W. Sun M. H. Qiu Y. Weng W. J. (2016). Sagittal alignment of the spine-pelvis-lower extremity axis in patients with severe knee osteoarthritis: a radiographic study. Bone Jt. Res. 5, 198–205. 10.1302/2046-3758.55.2000538 27231331 PMC4921054

[B104] Wang S. Li W. Zhang P. Wang Z. Ma X. Liu C. (2022). Mechanical overloading induces GPX4-regulated chondrocyte ferroptosis in osteoarthritis *via* Piezo1 channel facilitated calcium influx. J. Adv. Res. 41, 63–75. 10.1016/j.jare.2022.01.004 36328754 PMC9637484

[B105] Wang N. Lu Y. Rothrauff B. B. Zheng A. Lamb A. Yan Y. (2023). Mechanotransduction pathways in articular chondrocytes and the emerging role of estrogen receptor-α. Bone Res. 11, 13. 10.1038/s41413-023-00248-x 36869045 PMC9984452

[B106] Wang J. Ushirozako H. Yamato Y. Hasegawa T. Yoshida G. Banno T. (2023). Why does knee flexion in the standing position occur? Spinal deformity or knee osteoarthritis. J. Orthop. Surg. 31, 10225536231169575. 10.1177/10225536231169575 37039267

[B107] Wink A. E. Gross K. D. Brown C. A. Guermazi A. Roemer F. Niu J. (2017). Varus thrust during walking and the risk of incident and worsening medial tibiofemoral MRI lesions: the multicenter osteoarthritis study. Osteoarthr. Cartil. 25, 839–845. 10.1016/j.joca.2017.01.005 28104540 PMC5473434

[B108] Wink A. E. Gross K. D. Brown C. A. Lewis C. E. Torner J. Nevitt M. C. (2019). Association of varus knee thrust during walking with worsening WOMAC knee pain: the multicenter osteoarthritis study. Arthritis Care Res. 71, 1353–1359. 10.1002/acr.23766 PMC6430708 30242985

[B109] Wipperman M. F. Lin A. Z. Gayvert K. M. Lahner B. Somersan-Karakaya S. Wu X. (2024). Digital wearable insole-based identification of knee arthropathies and gait signatures using machine learning. eLife 13, e86132. 10.7554/eLife.86132 38686919 PMC11152572

[B110] Xian M. Lin J. Lu M. Lin Y. Li Z. Huang G. (2025). Proximal-dominant knee-ankle coordination compensations in football players with chronic ankle instability under high load. J. Exp. Orthop. 12, e70443. 10.1002/jeo2.70443 41103912 PMC12527221

[B111] Xie J. Li S. Song Z. Shu L. Zeng Q. Huang G. (2024). Functional monitoring of patients with knee osteoarthritis based on multidimensional wearable plantar pressure features: cross-sectional study. JMIR Aging 7, e58261. 10.2196/58261 PMC11629041 39586093

[B112] Yoon J. Moon S. W. (2024). Impacts of asymmetric hip rotation angle on gait biomechanics in patients with knee osteoarthritis. Knee Surg. Relat. Res. 36, 23. 10.1186/s43019-024-00226-5 39004757 PMC11247852

[B113] Yoon J.-Y. Moon S. W. (2026). Gait asymmetry in arm swing and foot progression angle of knee osteoarthritis. J. Clin. Med. 15, 5360. 10.3390/jcm15145360 42513275 PMC13410256

[B114] Zhang L. Liu G. Han B. Yan Y. Fei J. Ma J. (2021). A comparison of dynamic and static hip-knee-ankle angle during gait in knee osteoarthritis patients and healthy individuals. Appl. Bionics Biomech. 2021, 6231406–6231411. 10.1155/2021/6231406 34853606 PMC8629624

[B115] Zhang H. Chen Y. Jiang H. Yan W. Ouyang Y. Wang W. (2023). Comparison of accuracy for hip-knee-ankle (HKA) angle by X-ray and knee motion analysis system and the relationships between HKA and gait posture. BMC Musculoskelet. Disord. 24, 452. 10.1186/s12891-023-06437-3 37270561 PMC10239101

[B116] Zhang Y. Tawiah G. K. Wu X. Zhang Y. Wang X. Wei X. (2023). Primary cilium-mediated mechanotransduction in cartilage chondrocytes. Exp. Biol. Med. 248, 1279–1287. 10.1177/15353702231199079 PMC10625344 37897221

[B117] Zhang Y. Killen B. A. Mohout I. Willems M. Luyten F. P. Verschueren S. (2025a). Cartilage mechanical responses during gait as *in silico* biomarkers for medial knee OA progression. Sci. Rep. 15, 35303. 10.1038/s41598-025-19371-2 PMC12511356 41068265

[B118] Zhang Y. Zhuang H. Ren X. Zhou P. (2025b). Implications of mechanosensitive ion channels in the pathogenesis of osteoarthritis: a comprehensive review. Front. Cell Dev. Biol. 13, 1549812. 10.3389/fcell.2025.1549812 PMC12078208 40376614

[B119] Zhang B. Pang Y. Bai Y. Yin S. Yan Y. Li X. (2026). The mediating role of sacral slope in the relationship between lumbar lordosis and knee alignment in knee osteoarthritis: an imaging study. Front. Bioeng. Biotechnol. 14, 1760345. 10.3389/fbioe.2026.1760345 41800181 PMC12963331

